# Species information in whistle frequency modulation patterns of common dolphins

**DOI:** 10.1098/rstb.2021.0046

**Published:** 2021-10-25

**Authors:** Julie N. Oswald, Sam F. Walmsley, Caroline Casey, Selene Fregosi, Brandon Southall, Vincent M. Janik

**Affiliations:** ^1^ Sea Mammal Research Unit, Scottish Oceans Institute, University of St Andrews, Fife KY16 8LB, UK; ^2^ Southall Environmental Associates, 9099 Soquel Drive, Suite 8, Aptos, CA 95003, USA; ^3^ Long Marine Laboratory, Institute of Marine Sciences, University of California Santa Cruz, 1156 High Street, Santa Cruz, CA 95064, USA

**Keywords:** common dolphin, *Delphinus delphis*, *Delphinus bairdii*, whistles, vocal learning, species recognition

## Abstract

The most flexible communication systems are those of open-ended vocal learners that can acquire new signals throughout their lifetimes. While acoustic signals carry information in general voice features that affect all of an individual's vocalizations, vocal learners can also introduce novel call types to their repertoires. Delphinids are known for using such learned call types in individual recognition, but their role in other contexts is less clear. We investigated the whistles of two closely related, sympatric common dolphin species, *Delphinus delphis* and *Delphinus bairdii*, to evaluate species differences in whistle contours. Acoustic recordings of single-species groups were obtained from the Southern California Bight. We used an unsupervised neural network to categorize whistles and compared the resulting whistle types between species. Of the whistle types recorded in more than one encounter, 169 were shared between species and 60 were species-specific (32 *D. delphis* types, 28 *D. bairdii* types). *Delphinus delphis* used 15 whistle types with an oscillatory frequency contour while only one such type was found in *D. bairdii*. Given the role of vocal learning in delphinid vocalizations, we argue that these differences in whistle production are probably culturally driven and could help facilitate species recognition between *Delphinus* species.

This article is part of the theme issue ‘Vocal learning in animals and humans’.

## 1. Introduction

Identifying the selective advantage of signals is one of the main challenges when studying animal communication. An open-ended vocal learner, one that can learn new vocalizations throughout life, can increase communicative complexity by adding new call types to its repertoire [1], but the advantages of large or changing repertoires are not always clear. Bird species with large song repertoires, for example, are not necessarily more successful breeders than those with small repertoires [2]. Similarly, it is unclear why some vocal learners incorporate calls from other species into their repertoires. Often the assumption is that new call types allow the communication of new content. Why vocal learners introduce novel signals into their repertoires and the function of such signals are among the most intriguing topics in animal communication.

Delphinids have long been recognized as an interesting taxon for the study of how learned signals are used. They have advanced cognitive skills as well as a large vocal repertoire which can be modified by vocal learning through life [3]. They also do not produce song [4] and live in complex social networks [5]. Thus, their vocal repertoire is used in more direct social interactions not involving song and is comparable to the use of hand signals in bonobos [6] or social calls in parrots [7]. The communication signals produced by delphinids are generally divided into two categories: whistles and burst pulses. Whistles are narrowband, frequency modulated sounds that range in duration from several tenths of a second to several seconds. Burst pulses are clicks trains with very short (less than 10 ms) inter-click intervals that make individual clicks indistinguishable to human ears [8]. To date, only a few delphinid communication calls have been assigned a clear function or meaning [9]. Specific food calls have been reported for delphinids such as bottlenose dolphins (*Tursiops truncatus*) [10] and killer whales (*Orcinus orca*) [11], but it has been suggested that these evolved to manipulate prey behaviour and that the common attraction by conspecifics to these sounds is a by-product of their occurrence when fish are present. Many other studies have addressed broader contexts such as foraging or socializing and have therefore only found general features of communication calls (e.g. maximum or minimum frequency) related to context (e.g. [12–14]).

The most detailed information on the function of specific call types comes from identity signalling. While relevant behavioural contexts can be challenging to parse, the identity of an individual animal provides an opportunity to study call function in a clear and unchanging context. Bottlenose dolphins develop individually distinctive signature whistles that convey identity of the caller [15], and other individuals copy such signatures to address the signature owner [16]. A recent study on trained bottlenose dolphins found that animals within a training group also shared a non-signature call type, possibly as a group identity signal, but the exact context in which it was used was unclear [17]. Another identity feature that has been studied widely in dolphin acoustic signals is what species an individual belongs to [18]. Species identity is broadcast using species-specific acoustic signals by animals as diverse as Túngara frogs (*Physalaemus pustulosus*) [19], damselfish (*Dascyllus albisella*) [20] and cowbirds (*Molothrus ater*) [21]. Previous studies to develop acoustic classifiers have found that delphinids can be classified to species with varying degrees of accuracy using general time and frequency parameters measured from their acoustic signals (e.g. [22,23]). However, the possible use of distinctive call types in delphinid species recognition has not been addressed so far.

Common dolphins off California offer a unique opportunity to study species signalling in a vocal learner. Like bottlenose dolphins they develop signature whistles [24,25], but they also live as two genetically distinct species along the California Coast (short-beaked common dolphins, *Delphinus delphis*, and long-beaked common dolphins, *Delphinus bairdii*) [26], suggesting that selection pressure may favour species-level acoustic differentiation to maintain reproductive isolation. General time-frequency variables measured from whistles produced by these two species are very similar [27], and so here we investigate whether their overall whistle modulation patterns carry species information.

## 2. Methods

### (a) Fieldwork

Continuous passive acoustic recordings were obtained from schools of short- and long-beaked common dolphins in the Southern California Bight, off the northern end of Catalina Island from 2017 to 2020. Three surface drifting acoustic instruments were placed in close proximity to schools of dolphins (within approx. 1 km and generally much closer to or within schools). We selectively focused on single-species schools, as opposed to mixed-species groups. For every recording event (or ‘encounter’), species identity (based on differences in morphology and pigmentation [28]) was determined using three methods. (i) Using aerial images obtained from an octocopter drone (UAS APO-42, Aerial Imaging Solutions, Old Lyme, CT, USA). Only high-quality images where physical features of animals could be easily identified were used for species identification. (ii) Through genetic sequencing of skin and blubber samples obtained from the focal group following standard biopsy techniques [29]. An average of 10 individuals was sampled per recording event. (iii) Visual observers on rigid-hulled inflatable boats near dolphins and at an elevated, shore-based theodolite station provided species identification, school size estimation and confirmation that no other cetacean species were in the area.

Each acoustic recording instrument consisted of a surface buoy with a submersible recording package (either SNAP model 2.0, Loggerhead Instruments, Sarasota, FL, USA or Soundtrap ST300, Ocean Instruments NZ, Auckland, New Zealand) that recorded via a single omni-directional calibrated hydrophone (SNAP: HTI-96-min, High Tech, Inc., Long Beach, MS, USA; frequency response 0.002–30 kHz ± 3 dB; SoundTrap: integrated hydrophone, frequency response 0.02–60 kHz ± 3 dB) suspended by a shock-mounted cable at a depth of 10 m. Five-minute files were continuously recorded throughout an encounter (16-bit resolution, no compression, 44.1 or 96 kHz sampling rate). Recordings were collected before, during and after mid-frequency active sonar signals in the context of a behavioural response study. To avoid bias, recording efforts for both species were balanced across these conditions.

### (b) Whistle extraction

Data from each of the buoys were reviewed aurally and visually using Raven 1.5 software (ravensoundsoftware.com) and whistles were selected for analysis from the buoy with the highest signal-to-noise ratio (SNR) relative to the other simultaneously deployed buoys. To avoid over-sampling smaller groups of animals or specific individuals, a maximum of 100 whistles were extracted from schools with fewer than 100 animals. For larger schools, the maximum number of whistles extracted was equal to the estimated group size for that school, up to a ceiling of 200 whistles. A random number generator was used to randomly select indexed times within each recording and the first whistle beginning after this time was chosen for analysis. If the chosen whistle did not meet the criteria for analysis, the following whistle was selected. Fundamental frequency contours were manually traced from whistles using the Real-time Odontocete Call Classification Algorithm (ROCCA, [30]) module in PAMGuard (www.pamguard.org, [31]). When a contour had been traced, ROCCA automatically extracted the contour time and frequency point from each 1024-point fast Fourier transform window as well as 56 contour variables, including variables that describe frequency (e.g. minimum and maximum frequency, frequencies at one-quarter and one-half of the duration), shape (e.g. slopes at various points along the whistle contour, number of inflection points and steps) and duration (see the electronic supplementary material, table A1 for a complete list of variables measured).

Only contours with limited overlap with other whistles and SNR greater than approximately 6 dB were extracted and measured. Groups of common dolphins frequently whistle concurrently, resulting in multiple, overlapping whistles that appear as a loud band of indistinguishable contours in a central frequency range (5–15 kHz) on a spectrogram. The number of whistles selected from these time periods was limited to a maximum of three so as not to over-select whistles at atypically low- or high-frequency ranges.

The recordings also contained repeated stereotyped whistles, possibly representing individually distinctive signature whistles [32]. As sets of signature whistles would be non-independent measures of species-wide *Delphinus* whistle characteristics, no more than two repeated stereotyped whistles of the same type were selected per 5 min recording file.

### (c) Contour categorization

Whistle contours were organized into types using ARTwarp, which employs an unsupervised neural network to group contours with similar frequency modulation patterns into categories and uses dynamic time warping to allow for differences in the duration of whistles [33]. To be placed into a category, a whistle had to have a similarity index (also known as a vigilance parameter) of 96% as compared to the reference contour for that category. The vigilance parameter was set to 96% based on previous studies demonstrating that signature whistles were accurately sorted into categories using this vigilance parameter [33] and that dolphins can distinguish between different signature whistle contours [34]. This suggests that categorizing whistles with a vigilance parameter of 96% will result in whistle types that are distinguishable by dolphins. ARTwarp was run once with all whistles pooled and no prior categorization.

ARTwarp-generated whistle categories (or types) that contained whistles recorded from both short- and long-beaked common dolphins were considered *shared whistle types*. Whistle types that were recorded from only one school were considered *single-encounter whistle types*, and those that were recorded from more than one school were considered *multiple encounter whistle types*. Whistle types that contained two or more whistles produced by only one species and were recorded from at least two schools were considered *species-specific whistle types*. ARTwarp whistle types were further labelled as *oscillatory whistles* if the contour shape (i) consisted of at least two cycles of similar magnitude with two or more maxima, (ii) the maximum and minimum of each cycle was separated by at least 1 kHz, and (iii) at least 50% of the contour exhibited this pattern (definition modified from Gruden *et al*. [35]). Oscillatory whistles were identified through visual inspection of ARTwarp reference contours and whistle contours traced using ROCCA.

### (d) Statistical analyses

#### (i) Evaluation of species differences relative to simulated data

Sub-sampling whistles from two species with identical vocal repertoires should result in the appearance of some species-specific signals simply by chance. A permutation test was used to evaluate whether the observed number of species-specific whistle types was an artefact of sub-sampling. In the permutation test, the species identity of whistles belonging to types recorded from two or more schools was randomly assigned over 1000 iterations. The resulting proportion of species-specific whistle types was calculated for each dataset and the *p*-value for a two-tailed significance test was calculated as the proportion of permutations with a higher or lower number of species-specific contour types than the true dataset.

#### (ii) Univariate comparisons

Mann–Whitney *U*-tests were used to compare the 50 continuous ROCCA-generated whistle variables across species (electronic supplementary material, table A1). The six categorical variables were compared with Pearson's χ^2^-tests. Statistical significance for these tests was evaluated using a Bonferroni-adjusted alpha value calculated as the standard alpha value divided by the number of tests (0.05/56, *α*_corrected_ = 8.9 × 10^−4^). Univariate comparisons of species-specific whistle types and single-encounter whistle types were conducted using one whistle from each type to avoid pseudoreplication.

#### (iii) Classification using random forest analysis

Random forest analysis was used to classify whistles to species using the *randomForest* package in R v. 4.0.2 [36]. A random forest is a collection of decision trees grown using binary partitioning of data. Each binary partition is based on the variable that produces the most homogeneous daughter nodes. Randomness is introduced into the tree-growing process by choosing the splitting variable from a random subset of variables at each node. Once the forest has been created, new observations are classified as the class with the most support or ‘votes’ from its constituent trees. The Gini Variable Importance Index is an output of the random forest analysis and is a relative measure of the degree to which each variable contributes to the model predictions [37].

In this analysis, each tree was grown using a bootstrapped subset of whistles. This allowed the quantification of predictive accuracy of the forest using ‘out-of-bag’ data. The default number of candidate predictor variables was used at each node (*mtry* parameter: the square root of the total number of variables). The number of trees was initially set to 10 000 but was increased by an order of magnitude (×10) until the rate of misclassifications stabilized. Unlike linear discriminant analysis, random forest classification does not make strict assumptions regarding the variance and distributions of the predictor variables. Accordingly, all whistle measurements extracted by ROCCA were included as predictive variables.

Several random forest models were run using different subsets of the whistles to investigate the role of species-specific whistles in species identification. Classification models were created using: all whistles (RF1), only species-specific whistle types that occurred in more than one encounter (RF2), only shared whistle types (RF3), and only single-encounter whistle types (RF4). The classification success of RF2 was compared to that of RF4 to determine whether performance was related to the presence of signature whistles. If the classification success of RF2 was owing to signature whistles of animals accidentally recorded from more than one encounter rather than to species-specific whistle types, then the performance of RF2 should not differ from performance when using whistles only recorded in single encounters (RF4) which would have signature whistles in them as well. For each random forest, sample sizes were matched between species by taking a random subset of the larger class equal to the size of the smaller class, to ensure that imbalances in number of whistles did not affect classification results.

## 3. Results

We analysed recordings made during 14 short-beaked common dolphin encounters and 10 long-beaked common dolphin encounters. In total, we included 1774 whistles: 872 from long-beaked common dolphins and 902 from short-beaked common dolphins (see the electronic supplementary material, table A2 for details). In the 96 kHz sampling rate dataset, 15 whistles had maximum frequency values exceeding the Nyquist frequency (22 kHz) of the 44.1 kHz sampling rate dataset. These whistles were omitted from our analyses.

**Figure 1.**
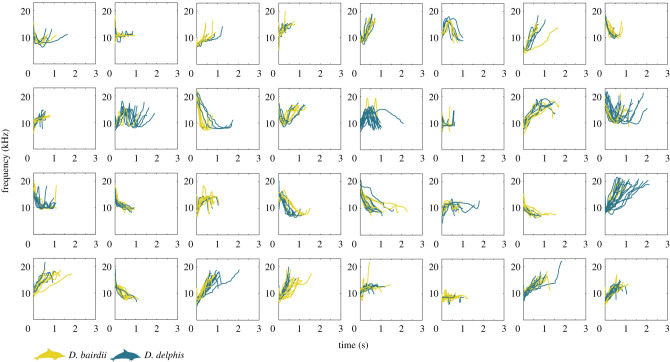
Examples of the most frequently occurring shared whistle types. Contours represent individual, traced whistles recorded from schools of short- and long-beaked common dolphins (*Delphinus delphis* and *Delphinus bairdii*, respectively) in the Southern California Bight. Species silhouettes were adapted from images available at phylopic.org (Chris Huh; creativecommons.org/licenses/by-sa/3.0/).

**Figure 2.**
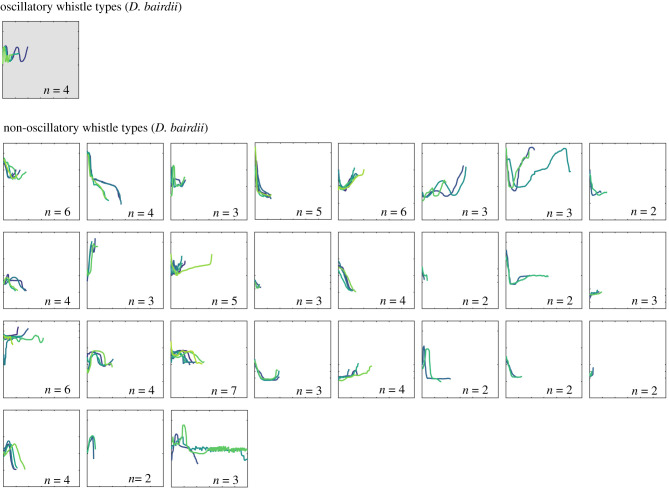
Whistle types produced exclusively by long-beaked common dolphins (*Delphinus bairdii*) that were detected during multiple encounters in the Southern California Bight. Colours represent individual whistle contours, with grey backgrounds designating oscillatory types. Axes on each box are the same, with *x*-axis showing 0–3 s and *y*-axis showing 0–23 kHz.

**Figure 3.**
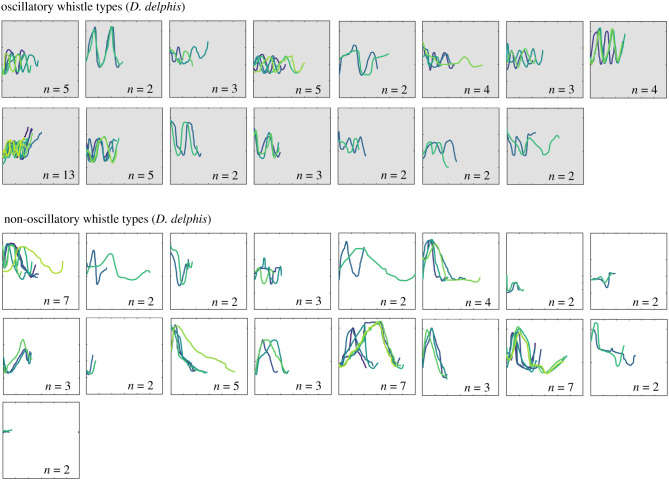
Whistle types produced exclusively by short-beaked common dolphins (*Delphinus delphis*) that were detected during multiple encounters in the Southern California Bight. Colours represent individual whistle contours, with grey backgrounds designating oscillatory types. Axes on each box are the same, with *x*-axis showing 0–3 s and *y*-axis showing 0–23 kHz.

ARTwarp grouped the 1774 traced whistles into 447 types. Most of these types (278 of 447, 62.2%) were exclusively detected during encounters of either short- or long-beaked common dolphins. Of these, 68.3% (190 of 278) represented just one whistle (electronic supplementary material, figure A1), and 10.1% (28 of 278) represented more than one whistle but were recorded from only one encounter. We expected these ‘singular’ types to include signature whistles of animals encountered once, as well as aberrant or rare vocalizations. After excluding whistle types recorded during a single encounter, we identified 169 shared whistle types (figure 1) and 60 species-specific whistle types; 28 for long-beaked common dolphins (figure 2) and 32 for short-beaked common dolphins (figure 3). Species-specific types were detected over a range of two to five encounters (mean 2.9 ± 1.0 s.d.) and across 1–3 years of recordings (mean 1.9 ± 0.59 s.d.). The permutation test confirmed that the true proportion of species-specific whistle types (26.2%, 60 of 229), was significantly greater than expected when the associated species of each whistle was shuffled at random (mean proportion = 15.5%, *p* = 0.001). Post-hoc visual assessment of whistle types revealed that a large proportion of short-beaked common dolphin species-specific types were oscillatory (15 out of 32 whistle types, representing 57 whistles), in stark contrast to long-beaked common dolphins, where only 1 out of 28 whistle types (representing four whistles) were oscillatory. Some of the single-encounter types that may represent signature whistles were also oscillatory (short-beaked *n* = 21 encounters, *n* = 22 whistles; long-beaked *n* = 24 encounters, *n* = 24 whistles). Oscillatory whistles also made up a very small proportion of shared whistle types (5 out of 169 types, representing 15 whistles).

Comparing all whistles recorded from each species, we found significant differences in 23 parameters, mostly relating to whistle duration, frequency and number of inflections (electronic supplementary material, tables A3 and A4). Fewer, though broadly similar, parameters were significantly different between species when only including whistles belonging to species-specific types: species-specific whistles produced by short-beaked common dolphins were longer, had larger frequency spreads, more frequency modulation and higher standard deviations of durations between inflections than those produced by long-beaked common dolphins (electronic supplementary material, tables A5, A6 and figure A2). By contrast, single-encounter whistle types were not differentiable by the same parameters, but showed significant differences in the mean of negative slopes, the shortest time between inflections, the number of inflections per unit time and the number of frequency steps per unit time (electronic supplementary material, tables A7 and A8).

The random forest models explored species-specificity of whistles further (table 1). Each random forest model contained 100 000 trees. The first random forest model including all whistles (RF1) discriminated whistles with low accuracy (68.7% overall correct classification, *n* = 1744 whistles). Overall classification accuracy increased significantly (Fisher's exact test, *p* < 0.0001) to 87.5% (*n* = 202 whistles) when only including species-specific types detected during multiple encounters (RF2). This model (RF2) also had a significantly higher correct classification score than the model that included only shared whistle types (RF3) (64.1%, *n* = 1238 whistles; Fisher's exact test, *p* < 0.0001). As expected, the classifier using whistles belonging to shared types classified species with the lowest accuracy. Despite being produced by only a single species, single-encounter types (RF4) provided a significantly lower species classification accuracy than species-specific types (RF2, Fisher's exact test, *p* = 0.0005) and was not significantly greater than RF1 (Fisher's exact test, *p* = 0.09). The electronic supplementary material, table A9 shows the 15 most important predictor variables for each random forest classifier, based on the Gini Variable Importance Index.

**Table 1.** Summary of classification accuracies for random forest classifiers including various subsets of short- and long-beaked common dolphin (*Delphinus delphis* and *D. bairdii*, respectively) whistles calculated using the *randomForest* package in R.

	number of whistles	classification accuracy (%)
RF1: all whistles	** **	** **
long-beaked	872	70.3
short-beaked	872	67.2
total	1744	68.7
RF2: whistles belonging to species-specific types (multiple encounters)		
long-beaked	101	88.1
short-beaked	101	87
total	202	87.5
RF3: whistles belonging to shared types		
long-beaked	619	62.7
short-beaked	619	65.4
total	1238	64.1
RF4: whistles belonging to single-encounter types		
long-beaked	111	77
short-beaked	111	71.2
total	222	74.5

## 4. Discussion

The results presented here demonstrate a clear difference in the use of oscillatory whistles between short- and long-beaked common dolphins, suggesting a role for frequency modulation patterns in species identification. A large number of oscillatory whistles is characteristic of short-beaked common dolphins, and long-beaked common dolphins appear to regularly use much simpler frequency modulation patterns. Many whistling delphinids, including common dolphins, encode individual identity information in the modulation patterns of their learned signature whistles [15] but all also produce a variety of non-signature whistles, the specific functions of which are relatively unknown [9]. This analysis of short-beaked common dolphin whistles identified 15 oscillatory whistle types, showing that it may be the occurrence of oscillations in the modulation pattern rather than one specific whistle type that carries species information. Thus, the encoding of species information differs from the way in which dolphins communicate individual identity, where each animal has one distinctive signature whistle type. It is unlikely that oscillatory whistle types represented signature whistles as species classification was most successful when only using multiple-encounter species-specific whistle contours. Using single-encounter whistles that probably included the signature whistles of many individuals, led to a much lower correct classification score than when using species-specific whistle types that occurred in multiple encounters in which signature whistles were less likely to occur (because they would only be included if the same animal was present in at least two encounters; table 1).

The use of specific modulation patterns or acoustic features to encode species identity has also been observed in other taxa. For example, three sympatric species of mouse lemurs in Madagascar produce tonal advertisement calls that do not differ significantly in frequency range or bandwidth but do have very different contour shapes. In playback experiments, individuals responded significantly more to conspecific advertisement calls than to heterospecific advertisement calls but did not exhibit a significant difference in response strength to other whistle types in their repertoires [38]. Similarly, in nestling brown-headed cowbirds, species recognition of conspecifics is triggered by a specific vocalization called the chatter [21]. In another example, Túngara frogs produce a two-part call consisting of a whine followed by a series of chucks. Playback experiments showed that it is the first 50 ms of the fundamental frequency of the whine that communicates species identity and the rest of the signal serves to make the call more attractive to females [39]. In a similar way, while frequency characteristics of the whistles of short- and long-beaked common dolphins overlap and make classification to species difficult, the presence of oscillations in the whistles of short-beaked common dolphins could serve as a species cue.

While oscillatory whistles were diagnostic of short-beaked common dolphins, the results of the random forest models showed that collectively, the species-specific whistle types identified by ARTwarp carry species information for both short- and long-beaked common dolphins. The random forest that included only multiple encounter, species-specific whistle types (RF2) had a significantly higher correct classification score than both the random forest that included all whistle types (RF1) and the random forest that included only shared whistle types (RF3) (table 1). However, some species-specific whistle types carry more species information than others. The random forest including only single-encounter whistle types (RF4) had a correct classification score that was higher than the random forests that included shared whistle types (RF1 and RF3), but significantly lower than the species-specific random forest (RF2), indicating that while single-encounter whistles carry species information, they do not carry as much as the multiple encounter, species-specific whistle types. This is probably owing to the presence of signature whistles in the single-encounter whistle types. Signature whistles carry individually specific information which led to higher correct classification scores than when looking at all whistle types or shared whistle types, but these whistles do not necessarily carry general species information, which led to lower correct classification scores than the multiple encounter, species-specific whistle types. In addition, different variables were important in RF2 and RF4 (electronic supplementary material, table A9), suggesting that species and individual information are carried in different whistle parameters. The single-encounter whistle variables that showed significant differences between species and were most important in the single-encounter random forest were those describing the shape of the whistles, such as slopes and the number and position of inflection points and steps (electronic supplementary material, tables A7–A9). This is consistent with previous work showing that it is whistle shape rather than voice characteristics that convey individual identity in signature whistles [40]. For species-specific whistle types, duration was the most important variable in the classifier, followed by variables describing the amount of frequency modulation in the whistles.

These results shed light on the possible functions of non-signature whistles and provide a new tool for the development of species classifiers for use in passive acoustic monitoring, which is particularly important for these species. *Delphinus delphis* and *D. bairdii* have only recently been recognized as two distinct species in the eastern North Pacific [41] and differentiation between the two has remained difficult. Consequently, current stock assessments and population trends for these two species in this region are unknown [42]. Additionally, detailed behavioural data for both short- and long-beaked common dolphins are lacking in the eastern North Pacific, which contributes to the difficulty of differentiating between these closely related species. In previous studies focused on developing species classifiers, the whistles of short- and long-beaked common dolphins have been difficult to discriminate owing to significant overlap in their time and frequency characteristics [27]. The ARTwarp analysis reported here showed that the whistle repertoires of these species can be divided into shared and species-specific whistle types. Using features measured from all whistles regardless of whistle type as input to classifiers obscures the species identity information that exists in species-specific whistle types and reduces classification success. Furthermore, the difference in the use of oscillatory whistles in short- and long-beaked common dolphins shows the value of exploring specific modulation patterns in addition to including more general time and frequency characteristics in classifiers.

Similarities and differences in the whistle repertoires of these two species are likely to have been caused by a variety of interacting factors such as behaviour, group size and social structure, and environment. Little is known about the behaviour and social interactions of these species and data on their distribution is limited. Short- and long-beaked common dolphins overlap in substantial areas of their ranges, however short-beaked common dolphins appear to range further offshore than long-beaked common dolphins [42]. These differences in distribution may have contributed to the development of different whistle repertoires adapted to different environments. It is also possible that the shared whistle types found between the two species in this study are remnants from the common dolphin repertoire before speciation and that oscillatory whistles became more dominant in short-beaked common dolphins as the two species evolved.

Learning influences vocal development in delphinids, increasing the potential for geographical variation and cultural specialization in how whistles are used. The species distinction between short- and long-beaked common dolphins is relatively clear in our study area [26] but not confirmed in other geographical regions [43], suggesting that populations in other geographical locations described as long-beaked common dolphins may share little with the population off California. The genetic and behavioural differentiation between short- and long-beaked common dolphins off California suggests that reproductive isolation between the two forms is more pronounced here than in other regions. Oscillatory whistles may have contributed to species isolation in these common dolphins, similar to the role of learned dialects in killer whale differentiation [44].

Oscillatory whistles have also been described for other dolphin species including bottlenose dolphins [45], spinner dolphins [35], pantropical spotted dolphins [35] and common dolphins in other areas [46–48]. While there are no studies describing the role of oscillatory whistles in the communication systems of these other species, their abundance across species makes it unlikely that they identify species more generally. Vocal learning makes shared whistle patterns a cultural trait, increasing the probability that oscillatory whistles have different functions across species and populations. The results reported here suggest that oscillatory whistle patterns of short-beaked common dolphins off California facilitate species recognition and potentially the speciation of common dolphins in a gene-culture coevolution scenario [49] specific to this geographical area. Future studies should investigate the role and context of oscillatory whistles in other regions and species to assess the diversity of their use.

## Data accessibility

The research data supporting this publication can be accessed at https://doi.org/10.17630/9305bcd2-b422-4496-8104-b9f856538c97 [50].
